# Supplementary material for: Toxicity of Carlina Oxide—A Natural Polyacetylene from the Carlina acaulis Roots—In Vitro and in Vivo Study
Source: Toxins (Basel). 2020 Apr 9;12(4):239. doi: 10.3390/toxins12040239 (PMC7232248; doi:10.3390/toxins12040239)
Supplement: Supplementary file 1 [file toxins-12-00239-s001.pdf]

Supplementary Material: Toxicity of Carlina Oxide – a Natural Polyacetylene from the *Carlina acaulis* Roots – *in vitro* and *in vivo* study

Artur Wnorowski, Sylwia Wnorowska, Kamila Wojas-Krawczyk, Anna Grenda, Michał Staniak, Agnieszka Michalak, Sylwia Woźniak, Dariusz Matosiuk, Grażyna Biała, Magdalena Wójciak, Ireneusz Sowa, Paweł Krawczyk and Maciej Strzemski

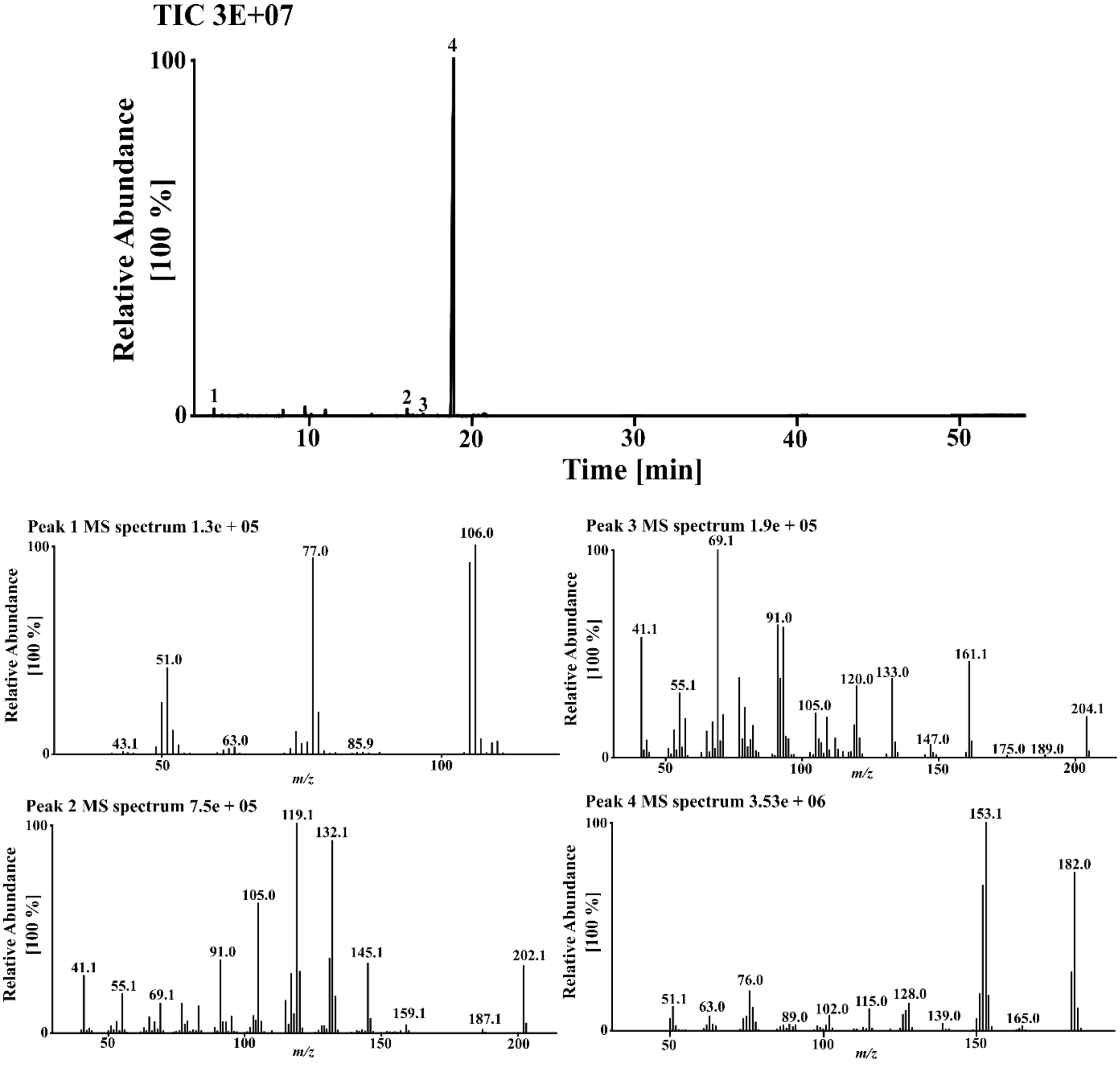

Figure S1. The total ion chromatogram and MS spectra obtained for the tested oil. 1: benzaldehyde, 2: *ar*-curcumene, 3: *β*-sesquiphellandrene, 4: carlina oxide.

Table S1. Chemical composition (% ± SD) of *Carlina acaulis* L. root essential oil.

Peak no.	Compound	RT (min)	RI	RI*	Mass data	Content %
1	benzaldehyde	4.11	960	952	106.0 (M+), 85.9, 77.0, 63.0, 51.0, 43.1	0.57 ± 0.002
2	ar-curcumene	16.01	1485	1481	202.1 (M+), 187.1, 159.1, 145.1, 132.1, 119.1, 105.0, 91.0, 69.1, 55.1, 41.1	0.56 ± 0.031
3	β-sesquiphellandrene	16.98	1526	1523	204.1 (M+), 189.0, 175.0, 161.1, 147.0, 133.0, 120.0, 105.0, 91.0, 69.1, 55.1, 41.1	0.16 ± 0.012
4	carlina oxide	18.87	1608	1613.1	182.0 (M+), 165.0, 153.1, 139.0, 128.0, 115.0, 102.0, 89.0, 76.0, 63.0, 51.1	96.2 ± 0.566
	Total					97.49

RT – retention times; RI - retention indices relative to C8-C20 alkanes on the DB-5MS column; RI* - literature retention indices [1]; M+ - molecular ion.

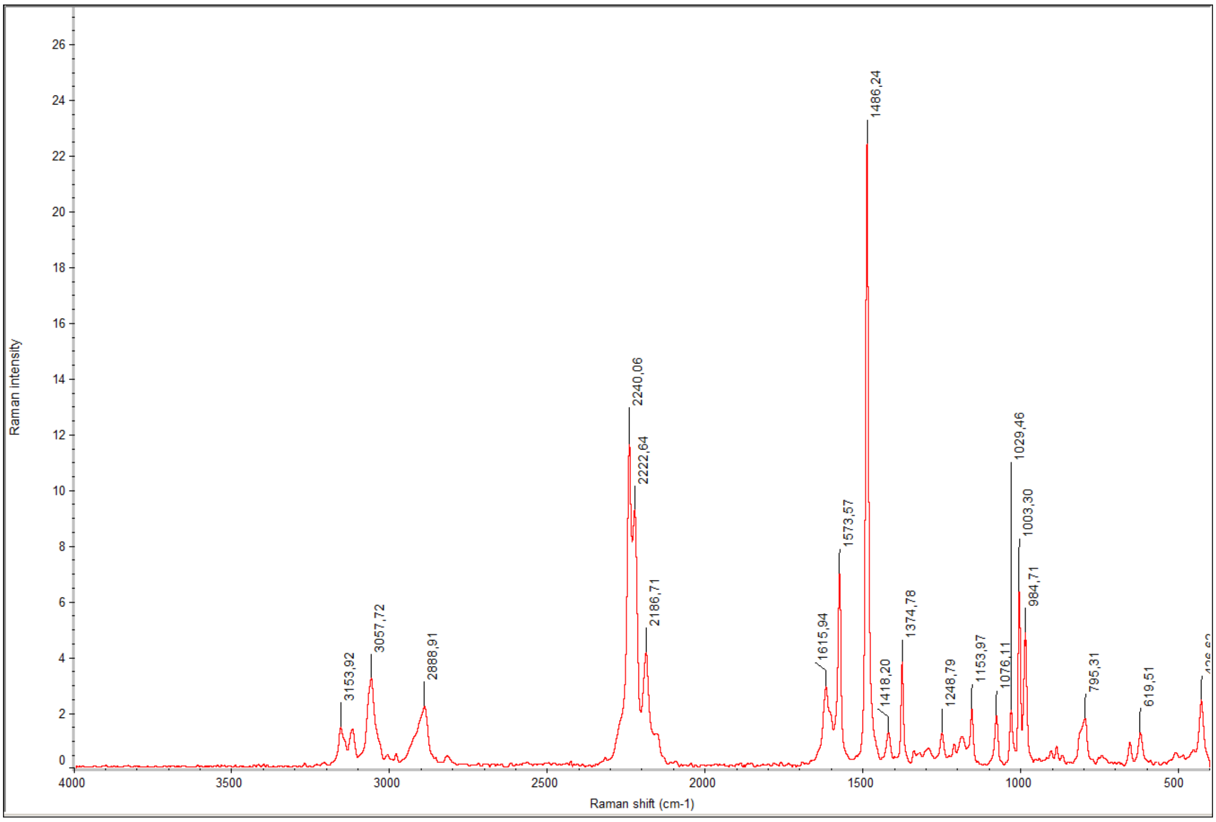

Figure S2. Raman spectrum of extracted essential oil.

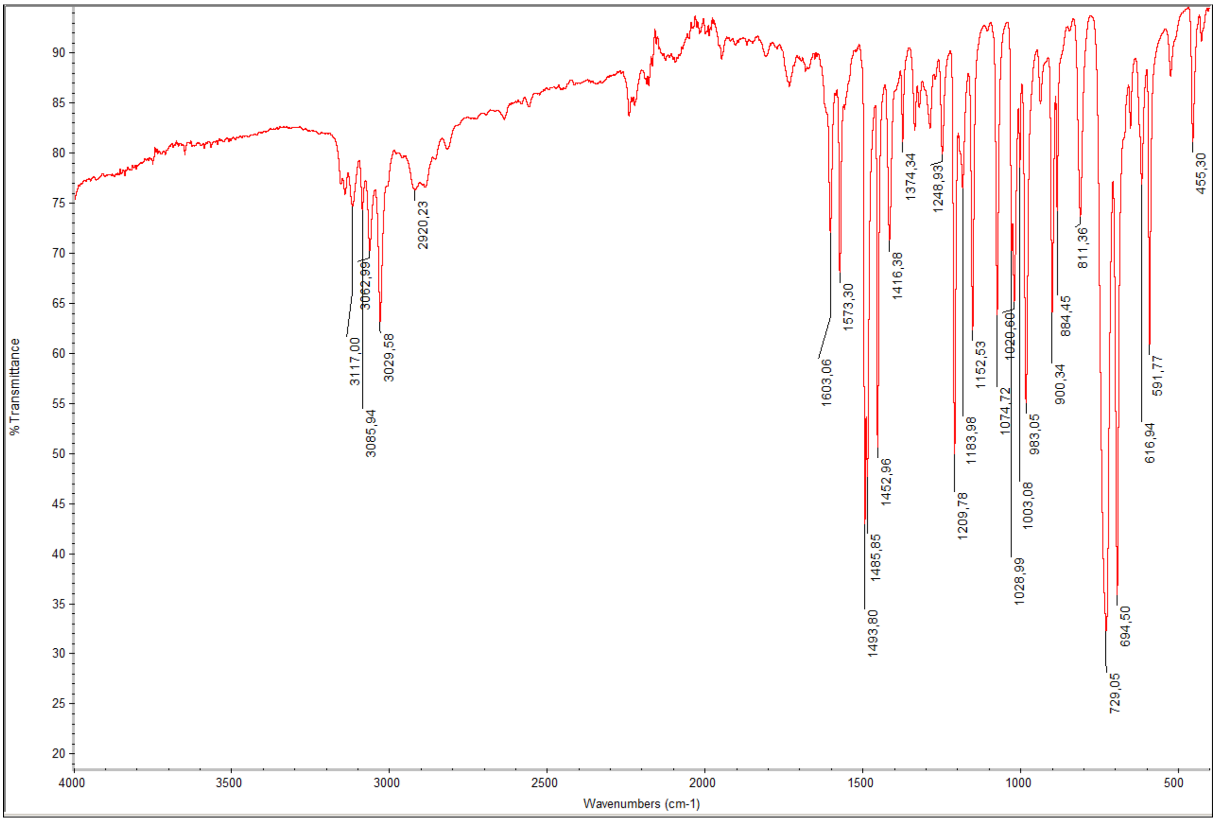

Figure S3. IR spectrum of extracted essential oil.

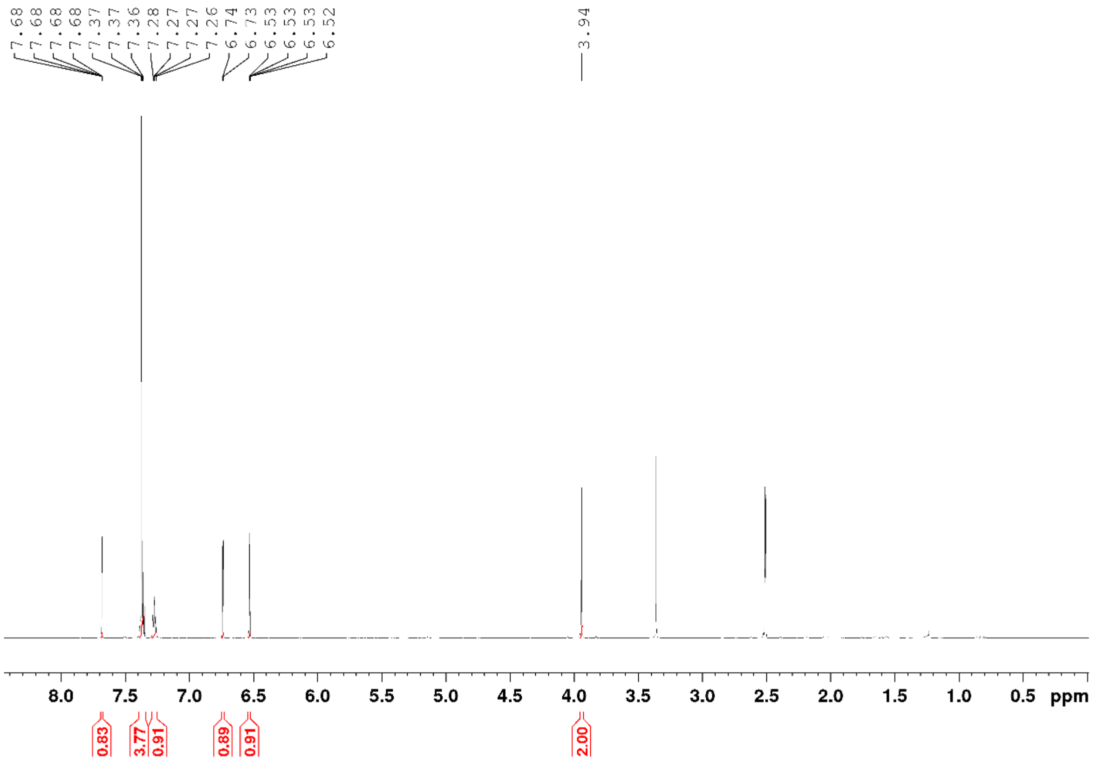

Figure S4. ^{1}H NMR (DMSO-d_6) spectrum of extracted essential oil.

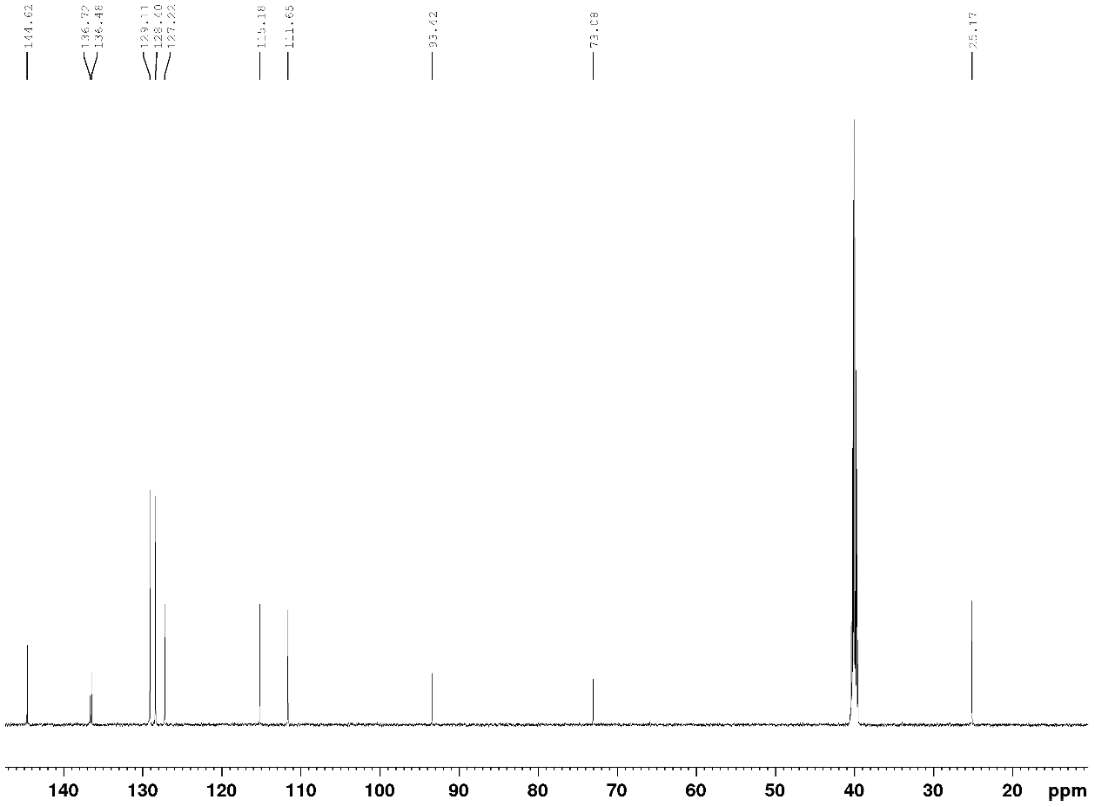

Figure S5. ^{13}C NMR (DMSO-d_6) spectrum of extracted essential oil.

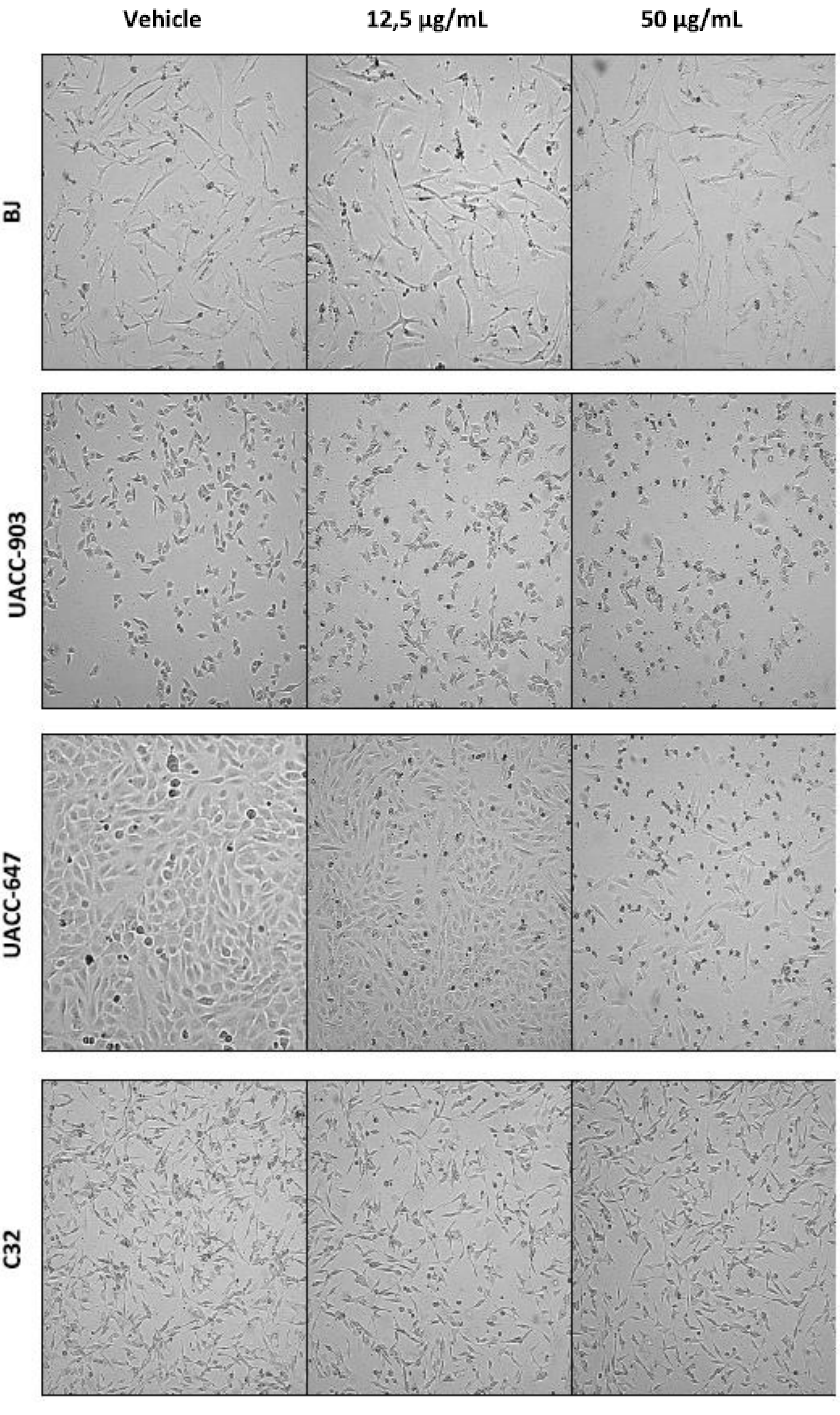

Figure S6. Representative microscopic images the BJ, UACC-903, UACC-647 and C32 cells exposed to either vehicle or carlina oxide (12,5 or 50 µg/mL) for 24 h.

References

1. Strzemski, M.; Wójciak-Kosior, M.; Sowa, I.; Agacka-Mołdoch, M.; Drączkowski, P.; Matosiuk, D.; Kurach, Ł.; Kocjan, R.; Dresler, S. Application of Raman spectroscopy for direct analysis of *Carlina acanthifolia* subsp. *utzka* root essential oil. *Talanta* **2017**, *174*, 633–637.
